# Recent advances in understanding and managing chronic pelvic pain in women with special consideration to endometriosis

**DOI:** 10.12688/f1000research.20750.1

**Published:** 2020-02-04

**Authors:** Elizabeth Ball, Khalid S Khan

**Affiliations:** 1 Department of Obstetrics and Gynaecology, The Royal London Hospital, Barts Health NHS Trust, London, UK; 2 Women’s Health Research Unit, Yvonne Carter Building, Queen Mary University of London, London, UK; 3 Centre for Maternal & Child Health Research, School of Health Sciences, City University of London, London, UK; 4 Department of Public Health, University of Granada, Granada, Spain

**Keywords:** chronic pelvic pain in women, endometriosis, idiopathic chronic pelvic pain

## Abstract

Chronic pelvic pain (CPP) in women is defined variably, but for clinical use it is cyclical or non-cyclical pain of at least 3–6 months’ duration. It has major impacts on individuals and society. There are both structural and idiopathic causes. Whereas CPP is not curable in many cases, it is treatable. The most promising approach is multidisciplinary patient-centered care including cause-directed therapy, lifestyle changes, talking therapies, meditation, acupuncture, and physiotherapy (this is not a complete list). One of the most common structural causes for CPP is endometriosis. This review investigates current scientific concepts and recent innovations in this field as well as for CPP in general.

## Introduction

Chronic pelvic pain (CPP) is defined as cyclical or non-cyclical pain of at least 6 months’ duration. Aspects of pain may include dysmenorrhea, dyspareunia, dysuria and dyschezia. Dysmenorrhea in isolation does not constitute CPP. CPP affects up to 24% of women worldwide ^1^. It accounts for 20% of gynecological clinic referrals ^2, 3^. It has a considerable impact on patients’ quality of life and their income, and annual costs to the NHS have been estimated at approximately £326 million ^4^ in addition to the costs to the public due to sick leave. One of the challenging issues is the long delay in women getting a diagnosis and accessing adequate care ^5^.

In some patients, an underlying structural pelvic pathology can be identified (e.g. endometriosis, adenomyosis, or chronic pelvic inflammatory disease with adhesions or hydrosalpinx), but often pain is idiopathic ^6^, meaning it is not due to a visible structural cause (e.g. bladder pain syndrome, irritable bowel syndrome, and pain memory), which describes the process of constant activation of the body’s pain perception system and applies to women with or without a structural disease such as endometriosis. It often occurs after an episode of acute pain, even if the painful stimulus has already been removed; mechanisms have been reviewed by Flor *et al*. ^7^. In fact, changes in the brain have been reported in endometriosis patients with CPP but not in asymptomatic women with endometriosis ^8^.

In many cases, the pathology is multifactorial ^9^. Follow up studies have shown that the surgical approach is frequently not curative. For instance, for endometriosis, 20–28% of patients do not experience a reduction in pain ^10, 11^ and some require another operation: 25.5% within 2 years and 40–50% after 5 years ^12^.

CPP is often resistant to surgical and medical treatment and appears to respond better to a multimodal, holistic approach rather than reliance on laparoscopy alone ^13, 14^. What is therefore required is an evidence base for aspects of a multidisciplinary approach with a focus on improving the patient’s quality of life, including self-management and complementary therapies, while also taking into account fertility plans. Like diabetes or hypertension, CPP is a chronic, idiopathic, and incurable but successfully treatable condition.

## Recent advances in the management of endometriosis

### Endometriosis definition

One of the commonest structural causes for CPP is endometriosis. Endometriosis is a chronic inflammatory condition affecting 6–10% of women of reproductive age, defined by the presence of endometrial-like tissue outside the uterus, commonly affecting the lining of the pelvis and the ovaries ^5^ and frequently causing subfertility and pain during periods, sexual intercourse, and defecation (dyschezia). Pain can be managed with painkillers, hormonal interventions, and surgical removal using the laparoscopic approach. There is a considerable emotional and financial cost to patients and society: estimates of total direct costs ranged from $1,109 (£ 850) per patient per year in Canada to $12,118 (£ 9,298) per patient per year in the USA. Indirect costs of endometriosis ranged from $3,314 (£ 2,542) per patient per year in Austria to $15,737 (£ 12,075) per patient per year in the USA ^15^.

### Delay in diagnosis

An average 7–9 years’ delay in accessing treatment for endometriosis in the UK ^16^ leads to unnecessary suffering from a condition that could be improved with appropriate management. Currently accurate non-invasive diagnostic tests or biomarkers are lacking. A recent James Lind Alliance Research Priority Setting Initiative for Endometriosis identified “improved treatment and care of women with endometriosis” and a “non-invasive clinical prediction model” as top research priorities ^17^.

A systematic search revealed no usable diagnostic algorithms to predict the disease successfully. However, an analysis of primary care records identified pain and menstrual symptoms occurring within the same year (odds ratio [OR] 6.5, 95% confidence interval [CI]: 3.9 to 10.6) and lower gastrointestinal symptoms occurring within 90 days of gynecological pain (OR 6.1, 95% CI: 3.6 to 10.6) as predictive of the diagnosis of endometriosis several years before the formal diagnosis ^18^.

Importantly, a normal ultrasound scan cannot rule out endometriosis and in the presence of symptoms suggestive of endometriosis can give false reassurance ^19^. Specialized ultrasound approaches have been described ^20^, which have an improved pick up rate.

### Relationship between symptoms and severity

Traditionally, it has been emphasized that the extent of endometriosis can be disproportionate to the level of pain (women with severe endometriosis may be suffering little pain and women with a small disease volume may have high pain scores). Although this may still hold true for some women, newer evidence indicates a relationship with more severe disease and higher scores of pain during periods, as identified in a meta-analysis that was carried out for the recent NICE guidelines on endometriosis ^19^. Severe endometriosis infiltrating the bladder, bowel, and ureter can be suspected on clinical grounds, although symptoms can vary individually ^19^. A pelvic assessment can point towards severe endometriosis (reduced organ mobility and tender nodularity in the posterior vaginal fornix, pain associated with pressure on the ovaries or uterine ligaments elicited during palpation, and endometriotic vaginal lesions visualised by examination with a speculum) ^19^.

With the recognition of the need for a multidisciplinary team approach, care for women with severe endometriosis has been centralized in the UK to foster the evolution of highly specialized units with gynecologists, urologists, bowel surgeons, fertility specialists, and specialist nurses providing integrated services ^19^. Similar to the care of gynecological cancer, the care for women with severe endometriosis has been centralized to endometriosis centers, which underlie external quality control and prospective data collection ^21^. Outcome measures for about 5,000 women with advanced endometriosis from a national database and have recently been reviewed, showing sustained significant reductions in all pain types associated with endometriosis maintained at 2 years’ follow up. There is a significant improvement in quality of life, which is also sustained at 2 years.

### Who gets better from surgery?

Laparoscopy can be diagnostic, therapeutic, or both. In the case of positive findings, a “see and treat approach” has been recommended as the gold standard ^22^. This approach is currently under scientific scrutiny for women with mild endometriosis. In addition, guidelines on endometriosis management have been systematically assessed and their methodology was deemed substandard ^23^.

Evidence from a randomized controlled trial (RCT) with placebo surgery control including 39 women with all stages of endometriosis has shown an improvement of symptoms in the treated group (16 of 20 [80%]) versus the placebo group (6 of 19 [32%]) ^10^. Quality of life measurements were also significantly improved 6 months after excisional but not after placebo surgery.

However, surgery does not reduce pain in 20–28% of patients ^10^. Secondary findings from observational single center studies indicate a graded response regarding pain reduction after endometriosis surgery, inversely related to disease severity ^11, 24, 25^. One RCT found pain symptoms improved after endometriosis surgery in significantly more patients with moderate and mild endometriosis (∼100% and ∼70%, respectively) than minimal disease (∼40%) ^11^. In two other studies, women with deep infiltrating endometriosis experienced more pain reduction from surgery than those with superficial endometriosis ^25^.

In order to determine which subgroup of women with CPP and mild forms of endometriosis benefit, or if there is any benefit at all for those women undergoing laparoscopic treatment, Horne *et al*. recently called for a trial randomizing such women to laparoscopy with and without treatment ^26^. An NIHR-sponsored trial, aiming to create an algorithm to predict the improvement in pain and quality of life after surgery using existing data, is underway (CRESCENDO NIHR PB-PG-0317-20018).

Whereas women with more severe endometriosis appear to have the best pain improvement after surgery, it has also been shown that the excision of endometriosis needs to be complete ^27, 28 ^and in particular there needs to be the removal of deeply infiltrating implants not just that of ovarian cysts ^29^.

Unfortunately, even with the full excision of endometriosis, women with severe forms require repeat surgery due to pain recurrence. Abbott *et al*. ^30 ^reported that if the revised American Fertility score (a grading system for endometriosis severity) was >70 points (indicating severe endometriosis), it was predictive of requiring further surgery. Interestingly, of the women who had further surgery, endometriosis was proven histologically in only 68%.

This points towards other causes at play that are responsible for ongoing pain which need to be addressed by other approaches. Reasons for residual pain can be endometriosis recurrence but also co-existent conditions associated with endometriosis including adenomyosis, irritable bowel syndrome, bladder pain syndrome, and pain memory.

### Postoperative prevention of pain recurrence

Evidence of the value of postoperative medical treatment to prevent pain recurrence is inconclusive. Reviewing the existing literature, Somigliana *et al*. ^31^ concluded that a short 3–6 months’ course of hormonal therapy with a GnRH agonist after surgery was of limited or no benefit for endometriosis in general and for deep peritoneal endometriosis in particular. On the other hand, they reviewed evidence indicating a beneficial effect of prolonged hormonal therapy after surgery for deep endometriosis. There may be a role for aromatase inhibitors, but more good-quality studies are required ^32^.

The value of postoperative adjuvant therapy may relate to the completeness of surgery. In a retrospective study of 93 patients ^28^, women with incomplete excision who received post-operative GnRH agonist had a post-treatment improvement of a 10 cm visual analogue scale (VAS) score similar to that of patients who had undergone complete excision (4.5±3.2 versus 5.6±3.9, *P* = 0.272), whereas in patients who had undergone complete excision there was no added benefit during an 18-month follow up period.

A health economics analysis based on historical data from 1,106 women with first diagnosis of endometriosis observed between 1979 and 200 ^33^ was used for a recent Chinese analysis ^34^. This analysis suggested a cost saving of over $6,000 per patient who received 6 months of postoperative treatment with a GnRH agonist. Given the recent technical advances in surgery and centralization of care, it can be speculated that current surgery leads more often to complete excision with a reduced cost benefit.

Pre-empt (NIHR ISRCTN97865475), a current RCT, has been designed to examine the role of progesterone-containing contraceptives in reducing recurrence after surgery. Participants are randomly allocated to take either long-acting progestogens (either as 3-monthly injections or as a coil, which is inserted into the womb, where it remains for 5 years) or long-term treatment with the oral contraceptive pill. Results are awaited.

## Are there effective holistic and psychological approaches to endometriosis and chronic pelvic pain, including self-management?

Given the portion of non-responders to surgery (reviewed by Horne ^26^) and the recurrence of pain, even if there is no recurrence of endometriosis in 23%, patients are calling for evidence-based approaches that do not require surgery or taking hormones (author’s focus group with patients from endometriosis UK, 2018). It is well known that endometriosis and CPP negatively impact mental health and quality of life, suggesting that affected women may have an increased risk of developing psychological suffering as well as of sexual problems ^35^ due to the presence of pain.

### Diet

By far the largest study on diet and endometriosis is based on the dataset of the Nurses’ Health Study (n = 3,800 with laparoscopically confirmed endometriosis) ^36^. Women consuming more than two servings per day of red meat had a 56% higher risk of endometriosis (95% CI: 1.22–1.99; *P* <0.0001) compared to those consuming one or fewer serving per week. Intakes of poultry, fish, shellfish, and eggs were unrelated to endometriosis risk.

A systematic review further reported ORs for the following foods and the presence of endometriosis: calcium intake OR: 0.99 (95% CI: 0.83–1.18), milk OR: 0.90 (95% CI: 0.65–1.23), eggs OR: 1.01 (95% CI: 0.81–1.28), bacon OR: 1.26 (95% CI: 0.60–2.65), and red meat OR: 1.26 (95% CI: 0.73–2.18) ^37^. Prospective trials investigating the effectiveness of dietary interventions are needed.

### Exercise

With endometriosis being both an inflammatory and an estrogen-dependent disease, it seems worthwhile to examine the effect of exercise, which is known to suppress both pathways. A systematic review of 3,355 women with endometriosis who had been doing recent physical activity and 4,600 cases who had been doing physical activity in the past reported that a pooled estimate of adjusted ORs for current exercise appeared to convey a significantly protective effect (OR: 0.69, CI: 0.53–0.89, Z = 2.83, *P* = 0.005), but the authors discuss their findings with a caveat because the overall estimates did not reach levels of significance ^38^.

### Acupuncture

A historic Cochrane systematic review of acupuncture in endometriosis ^39^ was able to include only a single study ^40^ with 67 participants randomized to acupuncture or Chinese herbal medicine. Dysmenorrhea scores were lower in the acupuncture group (mean difference –4.81 points, 95% CI: –6.25 to –3.37, *P* <0.00001) using the 15-point Chinese Medicine for Treatment of Pelvic Endometriosis scale.

Since then, a systematic review ^41^ of two sham-controlled RCTs and a retrospective study of 121 women with all stages of endometriosis ^41– 43 ^suggested a decrease in pain following acupuncture, although numerical data could not be meta-analyzed owing to the way outcomes were reported.

A further systematic review included two placebo-controlled RCTs ^43, 44^ on acupuncture in endometriosis showing that the 56 included endometriosis patients had more pain reduction with acupuncture than placebo (RR: –1.93, 95% CI: 3.33 to 0.53, *P* = 0.007) ^45^. A well-designed RCT protocol for a forthcoming study is underway ^46^.

### Psychological interventions

Given the association with stress and a pro-inflammatory immune response in addition to the poorer mental health that can be associated with endometriosis, psychological approaches appear to be promising. A current systematic review of psychological and mind–body interventions for endometriosis with narrative synthesis due to the variety of study designs ^47^ identified three RCTs, the remaining nine being non-randomized.

Psychotherapy with somatosensory stimulation ^48^ including a combination of Chinese medicine, hypnotherapy, cognitive behavioral therapy, and mindfulness was delivered in sessions over 3 months (n = 35) compared to waitlist controls (n = 32). The intervention group had reductions in maximal global pain (mean group difference –2.1, 95% CI: –3.4 to –0.8, *P* = 0.002), average global pain (–2.5, 95% CI: –3.5 to –1.4, *P* <0.001), pelvic pain (–1.4, 95% CI: –2.7 to –0.1, *P* = 0.036), and dyschezia (–3.5, 95% CI: –5.8 to –1.3, *P* = 0.003) and improvements in physical quality of life (3.8, 95% CI: 0.5–7.1, *P* = 0.026) and mental quality of life (5.9, 95% CI: 0.6–11.3, *P* = 0.031).

In another study, 40 women were randomly divided into two groups: an intervention group of women who were allocated to hatha yoga sessions twice a week for 8 weeks (n = 28) and a control group of women who did not practice yoga (n = 12). Daily pain was significantly reduced in the yoga group compared with those who did not practice yoga ( *P* = 0.0007) ^49^.

The third study randomly assigned 100 consecutive Chinese endometriosis patients to a progressive muscle relaxation (PMR) group (n = 50) and a control group (n = 50). Over 12 weeks, both groups received one dose of depot leuprolide, and the PMR group received 12 weeks of additional PMR training. Anxiety levels and depression were measured with validated instruments. The PMR group showed significant improvement in state anxiety, trait anxiety, and depression after intervention ( *P* <0.05) ^50^.

There is growing interest in using mindfulness-based interventions, which have been shown to be effective in other types of chronic pain ^51^. One of the uncontrolled studies from the previous systematic review is that of Hansen *et al*. ^52^, who reported sustained long-term effects (6-year follow-up) of a 10-session mindfulness-based psychological intervention for a series of 10 women with endometriosis-related CPP and improved quality of life.

Mindfulness meditation taught and delivered by a smartphone application has been investigated in a three-arm RCT (n = 90 women with chronic pain with and without endometriosis) compared to PMR and treatment as usual. The publication of results is awaited ^53^.

## Recent advances in the management of chronic pain

Laparoscopy is a costly and invasive “gold standard’ to diagnose causes of CPP. The recently completed MEDAL study ^54^ on 291 women with CPP aimed to determine the proportion of women with CPP for whom MRI is accurate enough to replace laparoscopy following evaluation of their symptoms. The authors concluded that MRI scans are not sufficiently accurate to find the cause of CPP in women and should not replace laparoscopy.

A Cochrane review from 2014 ^55^, which included 13 publications of non-surgical interventions for the management of CPP, reported moderate-quality evidence to support progestogen as an option for CPP at the cost of side effects such as weight gain and bloating. Other interventions such as a comparison of goserelin with progestogen, gabapentin with amitriptyline, “reassurance ultrasound” versus “wait and see”, and writing therapy versus non-disclosure provided too low-quality evidence or was drawn from a single study only. Thus, no recommendations could be made, and the authors called for RCTs of other medical, lifestyle, and psychological interventions.

Similarly, another Cochrane review on oral contraception for the treatment of endometriosis-related pain ^56^ concluded that the limited evidence from two trials at high risk of bias provided insufficient evidence to make a judgement on the effectiveness of the combined oral contraceptive pill (COCP) compared with placebo.

To provide an effective oral treatment to alleviate pain in women with CPP in the absence of any obvious pelvic pathology, a double-blind placebo-controlled randomized multicenter clinical trial called GAPP is underway. A total of 300 women with CPP and a normal laparoscopy will be randomized to gabapentin or placebo and their treatment will be titrated over a 4-week period to a maximum of 2,700 mg or placebo equivalent and maintained at that dose for 12 weeks. Average and worst pain scores will be measured by validated questionnaires. The results are expected soon ^57^.

A review on CPP management would not be complete without the mention of the role of physiotherapy, including treatment of myofascial trigger points, pelvic floor relaxation, and biofeedback. However, it is difficult to examine these treatments as stand-alone interventions, and a recent systematic review called for well-conducted, larger trials ^58^.

Future research should be directed at helping to shorten the delay in making the diagnosis of endometriosis, involving primary and secondary care. Women who benefit most from surgery should be identified through systematic review of evidence, new RCTs, or analysis of existing data (such as CRESCENDO NIHR PB-PG-0317-20018). Lifestyle measures such as diet and exercise for CPP need to be prospectively examined in RCTs.

In conclusion, progress is being made in creating better awareness of endometriosis, identifying approaches to diagnose endometriosis earlier, and enabling women to access effective treatment. However, not all women with CPP with or without endometriosis will benefit from surgery, and a multidisciplinary patient-centered approach is needed. Whereas evidence for non-surgical approaches is increasing, more RCTs on which to base recommendations are needed.

## Abbreviations

CI, confidence interval; CPP, chronic pelvic pain; OR, odds ratio; PMR, progressive muscle relaxation; RCT, randomized controlled trial.
